# Effectiveness of BNT162b2 Vaccine against Omicron Variant Infection among Children 5–11 Years of Age, Israel

**DOI:** 10.3201/eid2904.221285

**Published:** 2023-04

**Authors:** Aharona Glatman-Freedman, Yael Hershkovitz, Rita Dichtiar, Alina Rosenberg, Lital Keinan-Boker, Michal Bromberg

**Affiliations:** Israel Center for Disease Control, Israel Ministry of Health, Ramat Gan, Israel (A. Glatman-Freedman, Y. Hershkovitz, R. Dichtiar, A. Rosenberg, L. Keinan-Boker, M. Bromberg);; Tel Aviv University School of Public Health, Tel Aviv, Israel (A. Glatman-Freedman, M. Bromberg);; Haifa University School of Public Health, Haifa, Israel (L. Keinan-Boker)

**Keywords:** COVID-19, 2019 novel coronavirus disease, coronavirus disease, severe acute respiratory syndrome coronavirus 2, SARS-CoV-2, viruses, respiratory infections, zoonoses, BNT162b2, vaccine effectiveness, children, Omicron variant, B.1.1.529, Israel

## Abstract

We assessed effectiveness of the BNT162b2 vaccine against infection with the B.1.1.529 (Omicron) variant (mostly BA.1 subvariant), among children 5–11 years of age in Israel. Using a matched case–control design, we matched SARS-CoV-2–positive children (cases) and SARS-CoV-2–negative children (controls) by age, sex, population group, socioeconomic status, and epidemiologic week. Vaccine effectiveness estimates after the second vaccine dose were 58.1% for days 8–14, 53.9% for days 15–21, 46.7% for days 22–28, 44.8% for days 29–35, and 39.5% for days 36–42. Sensitivity analyses by age group and period demonstrated similar results. Vaccine effectiveness against Omicron infection among children 5–11 years of age was lower than vaccine efficacy and vaccine effectiveness against non-Omicron variants, and effectiveness declined early and rapidly.

Use of the BNT162b2 vaccine (Pfizer-BioNTech, https://www.pfizer.com) among children 5–11 years of age was approved by the US Food and Drug Administration and the European Medicines Agency in October 2021 (*1*,*2*). The approval was given after a randomized clinical trial conducted by the manufacturer found vaccine efficacy of 90.7% (95% CI 67.7%–98.3%) against laboratory-confirmed symptomatic COVID-19 with onset of >7 days after the second dose among 2,268 children in this age group who were included in that trial (*3*). In Israel, the vaccine was approved for children 5–11 years of age on November 14, 2021 (*4*), and the vaccination campaign was rolled out on November 22, 2021 (*5*). The BNT162b2 vaccination regimen for children 5–11 years of age consists of two 10-μg doses administered 21 days apart (*3*). By February 15, 2022, a total of 215,707 children had received 2 doses (18.2% of children in this age group in Israel), and 308,813 had received 1 dose (26.1% of children in this age group in Israel.

Shortly after the vaccination campaign among children 5–11 years of age began in Israel, the B.1.1.529 (Omicron) SARS-CoV-2 variant was identified in South Africa (*6*) and rapidly spread throughout the world (*7*). The Omicron variant has been described as having 30 aa substitutions, 3 insertions, and 6 aa deletions within the spike protein (*8*). The first case of infection with the Omicron variant was reported in Israel on November 27, 2021 (*9*), and by January 10, 2022, >90% of the sequenced samples in Israel were identified as the Omicron variant (*10*). 

In this study, we evaluated BNT162b2 vaccine effectiveness (VE) against SARS-CoV-2 infection among Israeli children 5–11 years of age during the Omicron-predominant period, which consisted mostly of the BA.1 subvariant. VE represents the degree to which vaccine prevents disease in real-world use, compared with vaccine efficacy, which indicates a controlled trial scenario. Approval for this study was granted by the Israel MOH superior ethics committee (protocol CoR-MOH-081–2021).

## Methods

### Study Design

After the second vaccine dose was given as part of the vaccine campaign for children 5–11 years of age, we estimated VE among Israel resident children who had not been infected with SARS-CoV-2 (as documented by PCR or official rapid antigen test) before the study period. We applied a matched case–control study design to estimate VE against SARS-CoV-2 infection for days 8–14, 15–21, 22–28, 29–35, and 36–42 after receipt of the second BNT162b2 vaccine dose and compared the vaccination status of children positive for SARS-CoV-2 by PCR (cases) with that of children negative for SARS-CoV-2 by PCR (controls).

During the early stages of the SARS-CoV-2 pandemic, multiple testing sites for SARS-CoV-2 were established throughout Israel. Those sites were operated by health maintenance organizations or by government-appointed operators, and testing was free of charge. During the Omicron wave, both PCR and rapid antigen tests were used, and results were entered into a national SARS-CoV-2 tests database.

To conduct our analyses, we used 2 Ministry of Health (MOH) national databases: the SARS-CoV-2 tests database and the SARS-CoV-2 vaccine database. The data retrieved from the databases included BNT162b2 vaccination status, BNT162b2 vaccination dates, PCR test dates and results, age, sex, population group (ultra-Orthodox Jews, general [which included non–ultra-Orthodox Jews and non-Arab minorities], and Arabs, based on statistical geographic area of a child’s residence), socioeconomic status (based on statistical geographic area), hospitalization, and the most severe clinical status (severe/critical disease or death) of hospitalized children. Severity of disease was determined according to the National Institutes of Health guidelines (*11*). For both databases, the unique personal identity numbers were encrypted twice.

We extracted data for January 20–February 15, 2022 (evaluation period). Those dates were selected because of the predominance of the Omicron variant, which exceeded 97% (*10*) and was predominantly BA.1. We excluded from analysis children who were SARS-CoV-2-positive by PCR or rapid antigen test before the evaluation period.

For children who had >1 positive PCR result during the evaluation period, only the first positive test was included in the analysis. For children who had >1 negative PCR result during the evaluation period, only the first negative test was included in the analysis. We excluded from analysis children who had a positive rapid antigen test result before a PCR test during the evaluation period. Each child who became SARS-CoV-2–positive during the evaluation period (case) was matched with 1 SARS-CoV-2–negative child (control) by age group (5–7, 8–9, and 10–11 years of age), sex, population group, socioeconomic status (low, medium, and high), and epidemiologic week of PCR sampling. We developed a flow diagram of the PCR tests included in and excluded from VE analyses (Appendix Figure 1).

We calculated the number and percentage of hospitalizations, severe or critical illnesses, or deaths among the cases who were hospitalized within 14 days of sampling, according to vaccination status. The selection of 14-day follow-up was based on a histogram delineating the time from first SARS-CoV-2 PCR sampling to hospitalization (Appendix Figure 2). Our data did not allow distinction between SARS-CoV-2–positive children hospitalized for COVID-19 or for other reasons.

### Statistical Analyses

To estimate the BNT162b2 VE, we used the formula (1 – OR) × 100, in which OR (odds ratio) represents the odds of cases being vaccinated divided by the odds of controls being vaccinated. We used conditional logistic regression for the analyses.

We applied the matching process and the statistical model separately for each week since receipt of the second vaccine dose (i.e., days 8–14, 15–21, 22–28, 29–35, and 36–42 since the second vaccine dose). To evaluate the robustness of the VE estimates, we performed 2 sensitivity analyses. For the first analysis, to determine if age-specific differences in VE estimates existed, we divided the data by age group (5–8 and 9–11 years) and conducted the same analysis for each group separately. For the second sensitivity analysis, to determine if VE estimates varied with the dynamics of infection, we divided the data by period and conducted the same analysis for each period separately. Period 1, which occurred around the peak of the Omicron wave in Israel, lasted from January 20 through February 2, 2022 (14 days). Period 2, which occurred during the decline in new SARS-CoV-2 cases, lasted from February 3 through February 15, 2022 (13 days). We also calculated the mean and median intervals (in days) between the second vaccine dose and the positive SARS-CoV-2 result for study participants in periods 1 and 2. To perform statistical analyses, we used SAS Enterprise Guide 7.1 software (SAS Institute Inc., https://www.sas.com).

## Results

### Vaccination Campaign

**Figure 1:**
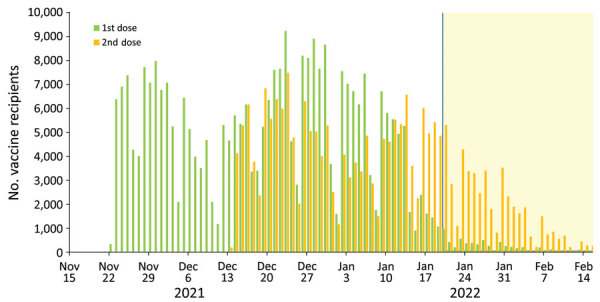
Progress of SARS-CoV-2 vaccination campaign among children 5–11 years of age by daily vaccine recipients, Israel. Green bars represent first-dose recipients; orange bars represent second-dose recipients; yellow highlighting represents the vaccine effectiveness evaluation period.

From November 22, 2021, through February 15, 2022, a total of 214,259 children 5–11 years of age received 2 BNT162b2 vaccine doses (Figure 1). That number represents 20.5% of Israel children 5–11 years of age in Isreal (*12*).

### VE against SARS-CoV-2 Infection

**Figure 2:**
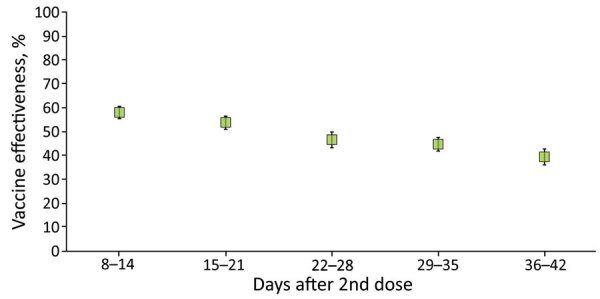
Vaccine effectiveness after second dose of BNT162b2 (Pfizer-BioNTech, https://www.pfizer.com) among children 5–11 years of age, Israel, January 20–February 15, 2022. The center of each symbol is the point estimate; error bars indicate 95% CIs.

During the evaluation period, we identified 78,541 SARS-CoV-2–positive eligible cases, of which 14,831 were second-dose recipients and 63,710 were unvaccinated. Case characteristics are summarized (Table 1). VE estimates after the second vaccine dose were 58.1% (95% CI 55.5%–60.6%) for days 8–14, 53.9% (95% CI 51.0%–56.5%) for days 15–21, 46.7% (95% CI 43.3%–49.9%) for days 22–28, 44.8% (95% CI 41.9%–47.6%) for days 29–35, and 39.5% (95% CI 36.1%–42.8%) for days 36–42 (Figure 2; Table 2).

**Table 1:** Characteristics of SARS-CoV-2 cases included in study of effectiveness of BNT162b2 vaccine against Omicron variant infection in children 5–11 years of age, January 20–February 15, 2022, Israel

Variable	Cases, no. (%), n = 78,541
Age, y	
5	9,783 (12.5)
6	11,310 (14.4)
7	11,529 (14.7)
8	11,599 (14.8)
9	11,468 (14.6)
10	11,562 (14.7)
11	11,290 (14.4)
Sex	
F	39,175 (49.9)
M	39,366 (50.1)
Population group	
Ultra-Orthodox Jews	2,194 (2.8)
General*	54,700 (69.6)
Arabs	21,647 (27.6)
Socioeconomic status	
Low	26,048 (33.2)
Medium	36,515 (46.5)
High	15,978 (20.3)

*Includes non–ultra-Orthodox Jews and non-Arab minorities.

**Table 2:** VE against SARS-CoV-2 infection among children 5–11 years of age, by time after the second vaccine dose, January 20–February 15, 2022, Israel*

Days since 2nd vaccine dose	Cases			Controls		Total cases, no.	Total controls, no.	VE (95% CI)
	Second-dose recipients, no.	Unvaccinated, no.		Second-dose recipients, no.	Unvaccinated, no.			
8–14	1,655	54,843		3,674	52,824	56,498	56,498	58.1 (55.5–60.6)
15–21	1,814	54,813		3,636	52,991	56,627	56,627	53.9 (51.0–56.5)
22–28	1,732	54,554		3,080	53,206	56,286	56,286	46.7 (43.3–49.9)
29–35	2,778	54,776		4,629	52,925	57,554	57,554	44.8 (41.9–47.6)
36–42	2,435	54,627		3,753	53,309	57,062	57,062	39.5 (36.1–42.8)

*VE, vaccine effectiveness.

### Sensitivity Analyses

**Figure 3:**
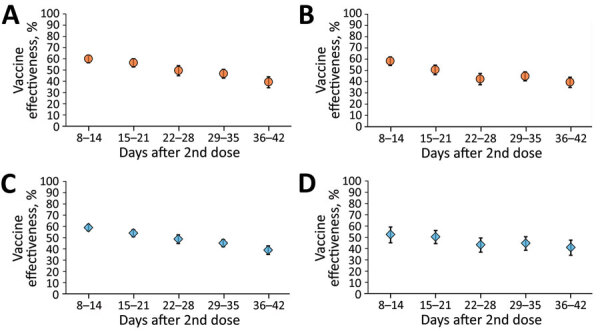
Sensitivity analyses for BNT162b2 (Pfizer BioNTech, https://www.biontech.com) vaccine effectiveness among children, Israel. A) Children 5–8 years of age, January 20–February 15, 2022; B) children 9–11 years of age, January 20–February 15, 2022; C) children 5–11 years of age, period 1 (January 20–February 2, 2022); D) children 5–11 years of age, period 2 (February 3–15, 2022). The center of each symbol is the point estimate; error bars indicate 95% CIs.

VE estimates for children in the 5–8- and 9–11-year age groups (Figure 3, panels A, B; Appendix Table) were similar to those found in the primary analysis (Figure 2; Table 2). Specifically, estimates of VE 8–14 days after the second vaccine dose were 60.0% (95% CI 56.5%–63.2%) for the 5–8-year age group and 58.4% (95% CI 54.4%–62.0%) for the 9–11-year age group (Appendix Table), compared with 58.1% (95% CI 55.5%–60.6%) for the primary analysis (Table 2). VE estimates 36–42 after the second vaccine dose were 39.4% (95% CI 34.3%–44.0%) for the 5–8-year age group and 39.6% (95% CI 34.8%–43.9%) for the 9–11-year age group (Appendix Table), compared with 39.5% (95% CI 36.1%–42.8%) for the primary analysis (Table 2).

Sensitivity analysis by period demonstrated that estimates of VE after the second vaccine dose for periods 1 and 2 (Figure 3, panels C, D; Appendix Table) were similar to those of the primary analysis (Figure 2; Table 2). Specifically, estimates of VE 8–14 days after the second vaccine dose were 59.0% (95% CI 56.1%–61.7%) for period 1 and 52.6% (95% CI 45.1%–59.1%) for period 2 (Appendix Table), compared with 58.1% (95% CI 55.5%–60.6%) for the primary analysis (Table 2). VE estimates 36–42 days after the second vaccine dose were 39.0% (95% CI 35.1%–42.7%) for period 1 and 41.1% (95% CI 34.0%–47.5%) for period 2 (Appendix Table) compared with 39.5% (95% CI 36.1%–42.8%) for the primary analysis (Table 2).

The mean and median days calculated for intervals between the second vaccine dose and the positive SARS-CoV-2 PCR among study participants were similar for both periods. Specifically, the mean (±SD) was 26.5 (±10.1) days for period 1 and 26.5 (±9.3) days for period 2, and the median was 28 days for period 1 and 26 days for period 2. The small difference in medians between periods was not significant (p = 0.53).

### Hospitalization, Severe/Critical Disease, and Death

Of the 78,541 SARS-CoV-2–positive children in the study, 102 (0.13%) were hospitalized within 14 days of their swab sample collection date (Table 3). A total of 93 (0.15%) were unvaccinated, and 9 had received 2 vaccine doses (0.06%) (Table 3). The vaccinated children were SARS-CoV-2 positive >8 days after the second vaccine dose. A total of 8 hospitalized children were defined as severely/critically ill, and no deaths were reported. All 8 (0.01%) severely/critically ill children were unvaccinated (Table 3).

**Table 3:** SARS-CoV-2–positive children 5–11 years of age hospitalized within 14 days of sampling, Israel

Total, no. (%), n = 78,541	Unvaccinated, no. (%), n = 63,710	Second-dose recipients, no. (%), n = 14,831	Variable
102 (0.13)	93 (0.15)	9 (0.06)	Hospitalized
8 (0.01)	8 (0.01)	0	Severe/critical illness*
0	0	0	Died

*Severe Illness: oxygen saturation (SpO2) <94% on room air at sea level, a ratio of arterial partial pressure of oxygen to fraction of inspired oxygen (PaO2/FiO2) <300 mm Hg, respiratory rate >30 breaths/min, or lung infiltrates >50% (*11*). Critical Illness: respiratory failure, septic shock, and/or multiple organ dysfunction (*11*).

## Discussion

We demonstrated that VE of the BNT162b2 vaccine against SARS-CoV-2 infection with the Omicron variant (predominantly BA.1 subvariant) among children 5–11 years of age was substantially lower than the 90.7% (95% CI 67.7%–98.3%) vaccine efficacy found in a clinical trial conducted before the Omicron variant emerged (*3*). Furthermore, our findings suggest the occurrence of an early decline in VE among children in this age group during the study period. This pattern of VE decline was found in the 5–8- and 9–11-year age groups, both of which received a smaller vaccine dose than that approved for persons ≥12 years of age. A similar decline in VE occurred during 2 time periods of the study, one representing the peak of SARS-CoV-2 cases and the other representing a decline in the number of cases.

VE against Omicron infection among children 5–11 years of age in Israel was also lower than the reported VE against infection with non-Omicron variants for other age groups after the second vaccine dose. Specifically, although reported VE against infection with non-Omicron (mostly Alpha) variants 15–21 days after the second vaccine dose was 96.8% (95% CI 96.1%–97.4%) among persons >16 years of age and 91.2% (95% CI 87.4%–93.8%) against infection with the Delta variant among persons 12–15 years of age (*13*,*14*), VE during the equivalent period in our study was 53.9% (95% CI 50.1%–56.5%).

Several groups reported VE estimates against the Omicron variant among children 5–11 years of age that were substantially lower than reported VE against non-Omicron variants. A study from 4 US states demonstrated that the adjusted VE against symptomatic and asymptomatic Omicron infection at 14–82 days after the second BNT162b2 vaccine dose among children 5–11 years of age was 31% (95% CI 9%–48%) (*15*). A study from Singapore demonstrated VE of 36.8% (95% CI 35.3%–38.2%) after 2 BNT162b2 vaccine doses (*16*). A previous study from Israel demonstrated a short-term (7–21 days after the second dose) BNT162b2 VE of 51% (95% CI 39%–61%) against infection and 48% (95% CI 29%–63%) against symptomatic disease (*17*).

However, a possible rapid decline of VE for this age group during an Omicron-predominant period was suggested in studies from 2 countries. A study from the United States demonstrated that VE against symptomatic SARS-CoV-2 infection among children 5–11 years of age was 60.1% (95% CI 54.7%–64.8%) at 2–4 weeks and 28.9% (95% CI 24.5%–33.1%) at 2 months after the second vaccine dose (*18*). A study from New York state also suggested that protection against Omicron infection declined rapidly after the second vaccine dose (*19*). A study from Italy demonstrated that adjusted VE against infection decreased from 38.7% (95% CI 37.7%–39.7%) at 14–28 days after the second BNT162b2 vaccine dose to 21.2% (95% CI 19.7%–22.7%) at 57–97 days after the second dose (the investigators did not consider days 0–14 after the second dose as full vaccination status) (*20*).

Studies that compared VE of 2 doses of mRNA vaccine against the Omicron and the Delta variants in older persons demonstrated that VE against Omicron is lower than VE against Delta. Those VE differences were demonstrated against SARS-CoV-2 infection (C.H. Hansen et al., unpub. data, https://www.medrxiv.org/content/10.1101/2021.12.20.21267966), symptomatic diseases (*21*), emergency department and acute care visits (*22*), hospitalizations (*23*), and severe outcomes (*24*). Administration of mRNA vaccine booster doses also resulted in lower VE against symptomatic diseases as well as fewer urgent care visits and emergency department visits for infection with Omicron than for infection with Delta (*21*,*22*).

Neutralization titers of the Omicron variant by serum from vaccine recipients and recovered persons who had been infected with non-Omicron variants were considerably reduced, or neutralization failed (*8*). Neutralization titers against the Omicron variant were boosted after the third vaccine dose but to a lesser degree than for other variants (*8*,*25*).

Among the advantages of our study, we provide weekly VE estimates during the evaluation period, whereas 2 other studies of children 5–11 years of age used longer intervals between VE estimates (*18*,*20*). The weekly VE estimates enabled us to demonstrate that the rapid VE waning started soon after the second BNT162b2 vaccine dose. In addition, we evaluated VE during a period when Omicron predominance exceeded 97%, thus minimizing the possibility of a Delta variant effect on estimation of VE.

Our study was limited by being unable to adjust VE estimates for the underlying conditions because of the nature of the MOH databases. We were also unable to estimate VE against symptomatic disease because of paucity of this data. The value of estimating VE against infection cannot be underestimated because the SARS-CoV-2 pandemic unveiled that a substantial portion of the population can be asymptomatic and still transmit the virus (*26*). In that regard, a recent study demonstrated that symptomatic as well as asymptomatic children can carry high amounts of live, replicating virus, which can serve as a potential reservoir for virus transmission (*27*). Another study reported that SARS-CoV-2 RNA loads among children with COVID-19 symptoms were comparable to those among asymptomatic children (*28*). An additional study limitation results from having only small numbers of children who were hospitalized or had severe/critical disease and no deaths, precluding us from estimating VE against those outcomes.

Evaluating VE only among children who were tested by PCR may result in selection bias. As the number of Omicron cases in Israel increased, the Israel MOH recommended PCR testing for persons >60 years of age and at high risk and that all other persons be tested by rapid antigen test instead (*29*). However, despite those instructions, which went into effect on January 7, 2022, PCR tests were performed also for residents <60 years of age, including children, in part because of long lines at official rapid antigen test–specific sites. Thus, the daily rate of PCR testing among vaccinated children remained >1.3% during the evaluation period. In that regard, estimating VE against influenza by using the test-negative case–control design has been based on testing only a small fraction of all patients with influenza-related signs/symptoms, and the sampling methods have varied from study to study. Thus, the possibility of selection bias exists also for influenza VE studies. Israel has had very robust national PCR testing for SARS-CoV-2; however, as the pandemic evolved, the number of persons tested (by PCR or antigen) has gradually declined. As such, similar to estimating VE against influenza, future SARS-CoV-2 VE studies will also have to rely on smaller fractions of the population. That the SARS-CoV-2 PCR testing policy in Israel was not strictly adhered to when official rapid antigen testing was introduced and the consistent results of our sensitivity analyses reduce the effect or likelihood of substantial selection bias in our study.

In conclusion, our findings indicate that, after 2 BNT162b2 vaccine doses, protection against infection with the Omicron variant among children 5–11 years of age was lower than protection reported against non-Omicron variants. Our study further suggests that protection against Omicron infection wanes rapidly among children in this age group. 

Appendix Additional information from study of effectiveness of BNT162b2 vaccine against Omicron variant infection in children 5–11 years of age, Israel.
