# Supplementary material for: Effectiveness of BNT162b2 Vaccine against Omicron Variant Infection among Children 5–11 Years of Age, Israel
Source: Emerg Infect Dis. 2023 Apr;29(4):771–7. doi: 10.3201/eid2904.221285 (PMC10045694; doi:10.3201/eid2904.221285)
Supplement: Appendix — Additional information from study of effectiveness of BNT162b2 vaccine against Omicron variant infection in children 5–11 years of age, Israel. [file 22-1285-Techapp-s1.pdf]

Article DOI: https://doi.org/10.3201/eid2904.221285

*EID cannot ensure accessibility for supplementary materials supplied by authors. Readers who have difficulty accessing supplementary content should contact the authors for assistance.*

# Effectiveness of BNT162b2 Vaccine against Omicron Variant Infection among Children 5–11 Years of Age, Israel

## Appendix

**Appendix Table.** Sensitivity analyses for vaccine effectiveness (VE) against SARS-CoV-2 infection in 5-11 year old children by age group and period, 20 January to 15 February 2022, Israel.

| | | Cases | | Controls | | | | |
|---|---|---|---|---|---|---|---|---|
| Sensitivity analysis group | Time since 2nd vaccine dose (Days) | 2nd dose recipients (N) | Unvaccinated (N) | 2nd dose recipients (N) | Unvaccinated (N) | Total cases (N) | Total controls (N) | VE (95% CI) |
| 5-8 yo* | | | | | | | | |
| | 8-14 | 853 | 33,102 | 1,994 | 31,961 | 33,955 | 33,955 | 60.0 (56.5 to 63.2) |
| | 15-21 | 904 | 33,102 | 1,936 | 32,070 | 34,006 | 34,006 | 56.6 (52.8 to 60.1) |
| | 22-28 | 824 | 32,967 | 1,564 | 32,227 | 33,791 | 33,791 | 49.6 (45.0 to 53.9) |
| | 29-35 | 1,265 | 33,041 | 2,240 | 32,066 | 34,306 | 34,306 | 46.9 (42.9 to 50.6) |
| | 36-42 | 1,087 | 32,899 | 1,704 | 32,282 | 33,986 | 33,986 | 39.4 (34.3 to 44.0) |
| 9-11 yo | | | | | | | | |
| | 8-14 | 767 | 21,721 | 1,683 | 20,805 | 22,488 | 22,488 | 58.4 (54.4 to 62.0) |
| | 15-21 | 920 | 21,643 | 1,717 | 20,846 | 22,563 | 22,563 | 50.6 (46.2 to 54.7) |
| | 22-28 | 929 | 21,510 | 1,514 | 20,925 | 22,439 | 22,439 | 42.4 (37.2 to 47.2) |
| | 29-35 | 1,466 | 21,750 | 2,370 | 20,846 | 23,216 | 23,216 | 44.8 (40.7 to 48.7) |
| | 36-42 | 1,348 | 21,681 | 2,063 | 20,966 | 23,029 | 23,029 | 39.6 (34.8 to 43.9) |
| Period 1 | | | | | | | | |
| | 8-14 | 1,362 | 43,852 | 3,059 | 42,155 | 45,214 | 45,214 | 59.0 (56.1 to 61.7) |
| | 15-21 | 1,339 | 43,681 | 2,715 | 42,305 | 45,020 | 45,020 | 54.1 (50.8 to 57.1) |
| | 22-28 | 1,160 | 43,486 | 2,150 | 42,496 | 44,646 | 44,646 | 48.8 (44.8 to 52.5) |
| | 29-35 | 2,159 | 43,713 | 3,604 | 42,268 | 45,872 | 45,872 | 45.2 (41.9 to 48.3) |
| | 36-42 | 1,904 | 43,608 | 2,915 | 42,597 | 45,512 | 45,512 | 39.0 (35.1 to 42.7) |
| Period 2 | | | | | | | | |
| | 8-14 | 294 | 10,927 | 586 | 10,635 | 11,221 | 11,221 | 52.6 (45.1 to 59.1) |
| | 15-21 | 491 | 11,056 | 923 | 10,624 | 11,547 | 11,547 | 50.5 (44.4 to 56.0) |
| | 22-28 | 575 | 11,008 | 949 | 10,634 | 11,583 | 11,583 | 43.5 (36.8 to 49.4) |
| | 29-35 | 586 | 11,036 | 993 | 10,629 | 11,622 | 11,622 | 44.8 (38.5 to 50.5) |
| | 36-42 | 533 | 10,961 | 858 | 10,636 | 11,494 | 11,494 | 41.1 (34.0 to 47.5) |

*yo –years old

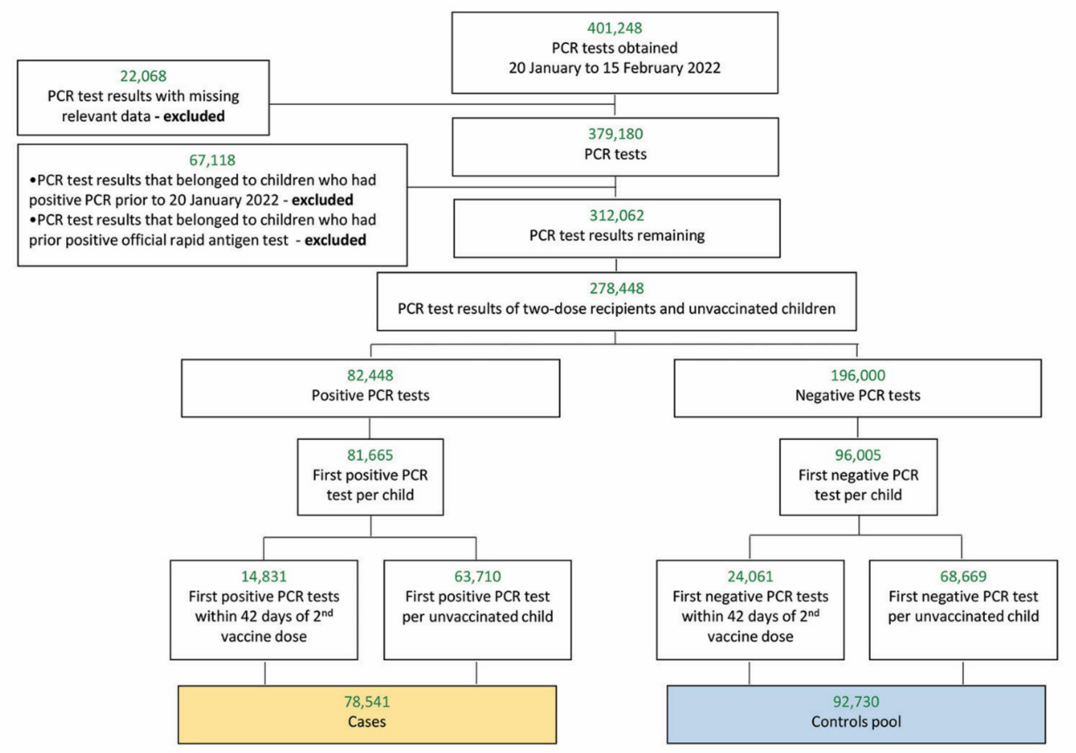


**Appendix Figure 1**. A flow diagram of PCR tests eligible for selecting cases and controls for matched case-control analysis.

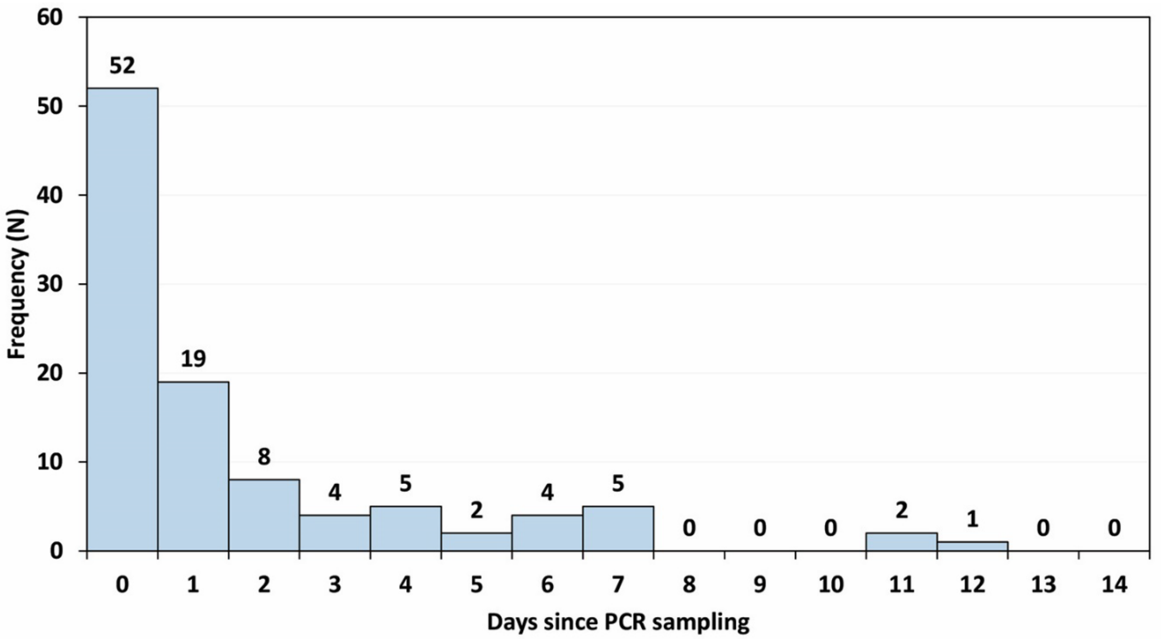


**Appendix Figure 2.** Time from first SARS-CoV-2 PCR sampling to hospitalization (N=102).
